# Locally Administrated Perindopril Improves Healing in an Ovariectomized Rat Tibial Osteotomy Model

**DOI:** 10.1371/journal.pone.0033228

**Published:** 2012-03-13

**Authors:** Xiong Zhao, Zi-xiang Wu, Yang Zhang, Ming-xuan Gao, Ya-bo Yan, Peng-chong Cao, Yuan Zang, Wei Lei

**Affiliations:** Department of Orthopeadics, Xijing Hospital, Fourth Military Medical University, Xi'an, People's Republic of China; Karolinska Insitutet, Sweden

## Abstract

Angiotensin-converting enzyme inhibitors are widely prescribed to regulate blood pressure. High doses of orally administered perindopril have previously been shown to improve fracture healing in a mouse femur fracture model. In this study, perindopril was administered directly to the fracture area with the goal of stimulating fracture repair. Three months after being ovariectomized (OVX), tibial fractures were produced in Sprague–Dawley rats and subsequently stabilized with intramedullary wires. Perindopril (0.4 mg/kg/day) was injected locally at the fractured site for a treatment period of 7 days. Vehicle reagent was used as a control. Callus quality was evaluated at 2 and 4 weeks post-fracture. Compared with the vehicle group, perindopril treatment significantly increased bone formation, increased biomechanical strength, and improved microstructural parameters of the callus. Newly woven bone was arranged more tightly and regularly at 4 weeks post-fracture. The ultimate load increased by 66.1 and 76.9% (p<0.01), and the bone volume over total volume (BV/TV) increased by 29.9% and 24.3% (p<0.01) at 2 and 4 weeks post-fracture, respectively. These findings suggest that local treatment with perindopril could promote fracture healing in ovariectomized rats.

## Introduction

Osteoporosis is characterized by a reduction in bone mass and the micro-architectural deterioration of bone tissue, resulting in bone fragility and an increase in susceptibility to fracture [1], [2], [3]. Although most anti-osteoporosis drugs have the ability to decrease the risk of osteoporotic fractures, fractures still occur in patients undergoing medical treatment. For this reason, the safety and efficacy of most anti-osteoporosis drugs for fracture healing have been analyzed to determine whether they should be ceased or continued after fracture formation [4], [5], [6], [7]. Moreover, it is well known that anti-osteoporosis drugs also have the ability to promote fracture healing.

The renin–angiotensin system (RAS) is an endocrine system that controls body fluids, electrolyte balance, and blood pressure [8]. The main effector peptide in this system is angiotensin II (Ang II), which is formed from angiotensin I (Ang I) by the angiotensin-converting enzyme (ACE), a key molecule in this system. The RAS has been an important target of antihypertensive drugs [9], particularly ACE inhibitors [10] and angiotensin receptor blockers (ARBs).

Previous studies have indicated that different components of the RAS have been found to be synthesized and active in osteoblasts and osteoclasts [11], [12], [13]. One study showed that Ang II encouraged bone resorption in osteoblast and osteoclast co-cultures [12]. The same effect was found after stimulation with Ang I and was prohibited by the ACE inhibitor moexiprilat. This result indicates that Ang II is generated by osteoblasts or osteoclasts through the conversion of Ang I by ACE. Accordingly, another study hypothesized that a local RAS in bone might play an important role in the regulation of bone metabolism [13]. Schurman et al. [14] showed that Ang II suppressed osteoblastic cell differentiation and bone formation in vitro. This effect was caused by specific binding of Ang II to the AT1 receptor [15]. Finally, recent studies have also demonstrated the expression of RAS components in osteoblasts and osteoclasts in vivo [11]. Further evidence for a potential role of the RAS in bone metabolism is derived from clinical studies. Patients treated with an ACE inhibitor showed an increased bone mineral density (BMD) and a reduced fracture risk [16], [17].

Up to now, the extensive research on ACE inhibitors has, to a large extent, been conducted on undisturbed bone; only one study has been conducted to investigate their effects on fracture healing [18]. Because perindopril affects bone metabolism locally through the bone cells, a question that arises is whether local delivery of this drug to the fracture would work.

The purpose of the present study was to test the hypothesis that locally-applied perindopril has the ability to promote fracture healing. In this study, tibial fractures were produced in OVX rats and subsequently stabilized with intramedullary wires. Perindopril was injected locally at the fractured site for a treatment period of days. Callus quality was evaluated at 2 and 4 weeks post-fracture.

## Results

An increased body weight was observed in all rats during the experimental period. No significant difference was found in body weight between the OVX+perindopril and the OVX+vehicle rats. No infection occurred at the fracture site.

Twelve weeks after either the OVX or sham operation, the mean tibial BMD value was 0.214±0.010 g/cm^2^ in the OVX group, which was significantly lower than the value of 0.265±0.015 g/cm^2^ in the sham group (P<0.05), confirming the establishment of the osteoporosis model.

### The effect of perindopril on the callus formation

**Figure 1:**
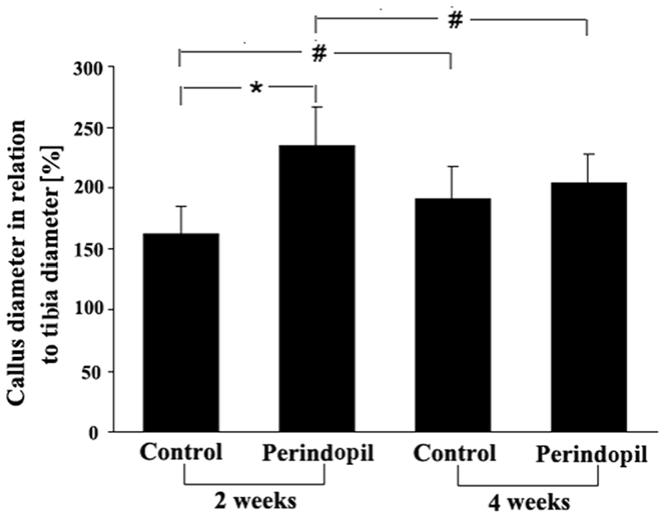
Radiological analysis of callus diameter in relation to the diameter of the tibia at the fracture site after perindopril treatment. All data are given as mean ± s.d. *P<0.05 versus corresponding values of the vehicle-treated controls. # P<0.05 versus corresponding values of specimens at 2 weeks post-fracture (n = 6 per group).

Two weeks post-fracture, radiological analysis showed that the diameter of the callus was significantly increased in the OVX+perindopril animals when compared with that of the OVX+vehicle controls (Fig. 1). However, at 4 weeks, OVX+vehicle controls exhibited an increase in callus diameter when compared with that of the controls at 2 weeks, whereas the size of the callus in OVX+perindopril animals was found to have decreased at this time point when compared with that of the OVX+perindopril animals at 2 weeks (Fig. 1). Thus, the difference in callus formation between the two groups observed at 2 weeks had disappeared by 4 weeks.

### The effect of perindopril on the biomechanical properties

The biomechanical properties of fractured tibiae are presented as ultimate load, energy absorption and stiffness (Table 1). As expected, the mechanical strength increased with time in both groups during the evaluation period. However, the fractured tibiae in the OVX+perindopril group showed significantly higher mechanical values than those in the OVX+vehicle OVX group, with the ultimate load increased by 66.1%, energy absorption by 53.2%, and stiffness by 51.7% at 2 weeks post-fracture (p<0.01). Similarly, the mechanical values of the OVX+perindopril group were increased compared with those of the OVX+vehicle group at 4 weeks post-fracture, with the ultimate load increased by 76.9%, energy absorption by 93.7% and stiffness by 63.9% (p<0.01).

**Table 1:** Biomechanical parameters in the fractured site of tibiae.

	OVX+vehicle	OVX+perindopril
2 weeks post-fracture (n = 6 per group)		
Ultimate load (N)	12.4±2.1	20.6±3.2**
Energy absorption (mJ)	7.9±1.4	12.1±2.8**
Stiffness (N/mm)	89.2±7.5	135.3±12.6**
4 weeks post-fracture (n = 6 per group)		
Ultimate load (N)	21.6±3.2	38.2±3.7**
Energy absorption (mJ)	14.2±2.1	27.5±3.4**
Stiffness (N/mm)	131.1±9.5	214.9±17.6**

Data are mean followed by standard deviation.

** P<0.01 vs OVX+vehicle group.

### The OVX+perindopril group showed more cartilage formation and greater newly forming trabeculae than the OVX+vehicle animals

**Figure 2:**
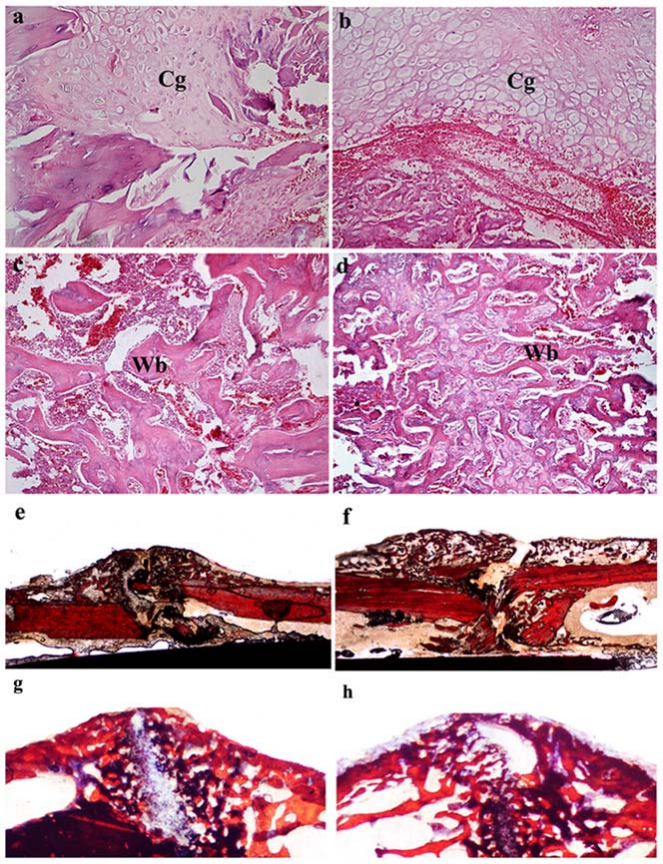
Histological presentations of OVX+vehicle (a, c, e, g) and OVX+perindopril (b, d, f, h) callus zones at 2 (a, b, e, f) and 4 (c, d, g, h) weeks post-fracture (n = 6 per group). Original magnifications: e, f: ×10; g, h: ×20; c, d: ×100; a, b: ×200. Cg, cartilage callus; Wb, woven bone.

At 2 weeks post-fracture, callus formation in the periosteum and endochondral ossification in the soft callus were recognized in both groups. Cartilaginous callus formation was visible in the OVX+perindopril animals, whereas less cartilage formation was visible in the OVX+vehicle animals. At 4 weeks post-fracture, compared with OVX+vehicle callus zones, new woven bone in OVX+perindopril animals arranged more tightly and regularly (Fig. 2). The OVX+perindopril group showed greater and denser newly forming trabeculae than the OVX+vehicle animals (Table 2). There was no sign of malignant transformations in any of the cell types in the callus areas in the perindopril-treated groups throughout the study period (Fig. 2).

**Table 2:** Micro-CT based histomorphometry of the fractured tibiae.

	OVX+vehicle	OVX+perindopril
2 weeks post-fracture (n = 6 per group)		
BV/TV(%)	39.8±4.5	51.7±5.7**
CsAr(mm^2^)	17.6±2.9	25.3±4.7**
4 weeks post-fracture (n = 6 per group)		
BV/TV(%)	61.7±5.1	76.7±6.2**
CsAr(mm^2^)	15.4±3.1	23.9±2.5**

BV/TV bone volume over total volume, CsAr average cross-sectional area.

Data are mean followed by standard deviation.

** p<0.01 vs OVX+vehicle group.

### The administration of perindopril significantly improved the histomorphometric parameters in the ovariectomized rat tibiae

**Figure 3:**
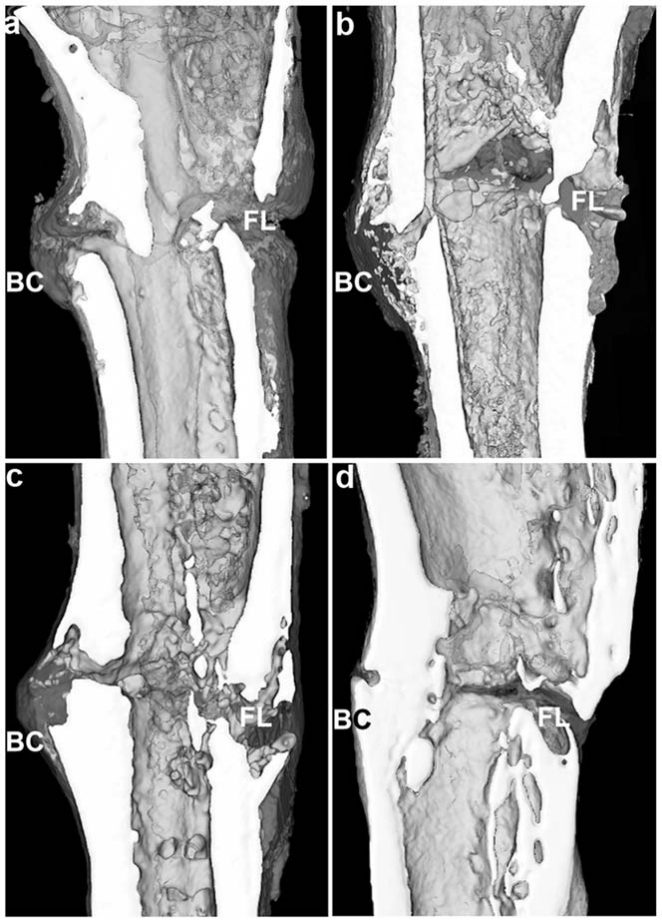
Micro-CT images from coronal cut aways via the central part of the 3-D reconstructions of fractured tibia at 2 (a, b) and 4 (c, d) weeks post-fracture (n = 6 per group); a, c for the OVX+vehicle group and b, d for the OVX+perindopril group. BC, bony callus; FL, fracture line.

From the three-dimensional reconstruction of the bony callus, a greater amount of bony callus was found in the OVX+perindopril group than in the OVX+vehicle group at both 2 and 4 weeks post-fracture, which demonstrates the anabolic effect of perindopril during the early period of fracture healing (Table 2). The fracture gap in both groups could be clearly seen at 2 weeks post-fracture. At 4 weeks, the fracture gap could be clearly observed in the OVX+vehicle group, while it was nearly invisible in the OVX+perindopril group (Fig. 3).

The results of the fractured tibiae (Table 2) from the Micro-CT evaluation were expressed as BV/TV and CsAr. At 2 weeks post-fracture, perindopril treatment significantly increased the values of BV/TV by 29.9% and CsAr by 43.8% (p<0.01) compared to the OVX+vehicle group. A similar trend was found at 4 weeks post-fracture, with BV/TV increased by 24.3% and CsAr by 55.2% (p<0.01). These quantitative results show that the administration of perindopril significantly improved the histomorphometric parameters in the ovariectomized rat tibiae.

## Discussion

In the current study, the results from radiological, biomechanical, histological, and micro-CT evaluations demonstrate that perindopril can promote fracture healing in OVX rats when injected subcutaneously into tissue in close proximity to the fracture. At both 2 and 4 weeks post-fracture, perindopril treatment induced increased callus, improved the callus microstructure and enhanced healing strength, and accelerated fracture healing compared to the untreated OVX group. To our knowledge, this is the first experimental study to demonstrate that locally applied perindopril can promote fracture healing in OVX rats, thereby confirming our hypothesis.

To the best of our knowledge, mechanical rehabilitation of the fractured bone is the key goal of fracture healing in clinics. Previous studies have indicated that osteoporotic fracture healing is a closely regulated process of regaining the textural geometry and mechanical features of the fractured bone [1], [25], [26], [27], [28]. For this reason, mechanical rehabilitation is not the culminate completion of fracture healing; geometrical and histological restoration are also important for the completion of natural fracture healing. In this study, we evaluated the effect of perindopril on the biomechanical features of the bony callus, and we also estimated its effect on the microstructure of the callus. The findings demonstrated that the mechanical parameters of the callus in the perindopril group were higher than those in the control group based on three-point bending tests. In addition, the microstructure of the callus in the perindopril group was superior to that of the control group as shown by micro-CT based quantitative and qualitative analysis and HE staining. Accordingly, these findings indicate that perindopril is beneficial for osteoporotic fracture healing by promoting the mechanical properties of the callus and facilitating the microstructure rehabilitation of the bony callus.

The ACE is a major constituent of the RAS, generating Ang II as the main effector molecule. Many experiments have indicated that Ang II can be synthesized by osteoblastic cells through the ACE and that Ang II affects osteoblastic cell functions through specific receptor binding [12], [13], [14], [15]. Ang II exerts its biological effects mainly through two receptors, the AT1 and AT2 receptors. Ang II has a large number of biological effects in different tissues and influences inflammation, angiogenesis, cell proliferation, cell differentiation and apoptosis [29]. Therefore, the RAS has been demonstrated to significantly influence tissue remodeling in various tissues [29]. Both AT1 and AT2 receptors induce apoptosis in different cell types [29], [30], [31]. ACE inhibition has also been shown to decrease apoptosis in various tissues [30], [32], [33].

Because ACE inhibitors are widely used in cardiovascular medicine, it is important to determine how these drugs may interfere with bone homeostasis. Clinical studies have shown that, under normotensive and hypertensive conditions, ACE inhibition significantly increased BMD, especially in the elderly [17]. Thus, normotensive patients with a I/I polymorphism and low ACE activity also had an increased BMD [16].

The unbalanced bone turnover is caused by extraordinary bone resorption and impaired bone formation, which could produce adverse effects on fracture healing in osteoporotic subjects. Anticatabolic drugs (i.e., bisphosphonates, estrogen, and salmon calcitonin) and anabolic agents (such as parathyroid hormone) have been used to treat osteoporotic fractures. Anticatabolic drugs based on the inhibition of bone resorption can result in an increased callus volume, BMD and biomechanical strength during the early healing period, and they might delay callus remodeling [34], [35]. Parathyroid hormone (1–34) has been demonstrated to improve cancellous bone healing at the site of an osteotomy [24], [36]. Thus, it is reasonable to study the effect of perindopril on fracture healing because the dual effects of the RAS on inhibiting bone resorption and promoting bone formation have been verified in vitro and in vivo [11], [37], [38], [39].

In a previous study, the effects of perindopril on bone fracture healing were examined. A femur fracture model was made and then stabilized with marrow nailing in a murine female fracture model, and perindopril was administrated systemically at a high dose (3 mg/kg/day) [18]. At 2 and 5 weeks after fracture, perindopril-treated animals showed significantly greater periosteal callus formation compared with controls. This study also demonstrated a greater torque to failure and a higher torsional stiffness after 2 and 5 weeks in perindopril-treated animals. In this study, perindopril was administered subcutaneously to the fracture site at a relatively lower dose, and increased callus area and enhanced fracture strength were found at 2 weeks post-fracture. Meanwhile, we also found increased fracture strength in the perindopril-treated fractures at 4 weeks post-fracture when the difference in callus size disappeared.

There are several limitations in this study. First, our fracture model was established based on a surgical osteotomy in contrast with a closed fracture model, the latter of which better describes common clinical fractures. However, this model enabled us to make a consistent fracture line, which is essential for making reliable and accurate evaluations. Second, perindopril was delivered by an invasive approach, which has the possibility of increasing the risk of infection. This problem can be solved if the drug is administered via an adequate carrier, delivering a local continuous dose. Nevertheless, the current study revealed that it might be possible to use ACE inhibitors to improve the healing of osteoporotic fractures.

In summary, our data show that local treatment with perindopril at a dose of 0.4 mg/kg/day promotes fracture healing of proximal tibiae in an ovariectomized rat model within a 4-week treatment period. Compared with the untreated OVX group, the perindopril treatment group showed an increase in callus quality and biomechanical strength, as well as improved callus microstructural parameters, without a negative effect on the natural fracture healing process. These animal fracture data suggest that the local application of perindopril could be a new option to improve the quality of fracture healing.

## Materials and Methods

### Animals

A total of 86, 3-month-old female Sprague–Dawley rats (purchased from the Experimental Animal Center of The Fourth Military Medical University, Xi'An, China) with an average weight of 250 g at the beginning of the study were used. The rats were maintained at 20°C on a 12-h light/dark cycle with free access to water and rat food containing 0.46% calcium and 0.38% phosphorus. All experimental procedures in animals were approved by the Ethics in Animal Research Committee of the Fourth Military Medical University (permission code 2010C00843).

### Fracture surgery

Three months after being ovariectomized (OVX) (n = 76) or sham-operated (n = 10) according to previous studies [19], osteopenia in the OVX rats was diagnosed using the Lunar iDXA scanner (GE Healthcare Lunar, USA) with the hand-regional high-resolution and small-animal scan mode as previously described [1]. The region of interest was defined as a longitudinal rectangle that was adjusted to cover the total right tibial area. General anesthesia for all operative procedures was achieved with intraperitoneal injections of 10% chloral hydrate (3.3 ml/kg). Then, in all animals, a simple transverse open fracture model was made in the proximal one-third of the right tibia, as previously reported [19], [20]. Briefly, a 2-cm longitudinal incision was made on the medial side of the knee joint. After careful dissection of subcutaneous tissues, a transverse osteotomy was made at the proximal one-third of the proximal tibia. Subsequently, the patella was deflected laterally, and a hole was drilled in the anterior intercondylar area. Then, the fracture fragments were contacted, and a stainless steel wire (1.0 mm in diameter) was inserted through a hole across the fracture ends. Soft tissues were sutured, and the animals received an intramuscular antibiotic and analgesic injection for three postoperative days. Each animal received antibiotics (penicillin, 0.80 million units, IM, BID) for three days postoperatively. Unrestricted activity was allowed after the anesthesia resolved. Among the 76 animals present at the beginning of the study, 4 rats died due to anesthesia and surgical trauma. The remaining 72 rats were randomly divided into four groups (n = 18 for each group).

### Pharmaceutical intervention

The perindopril powder was dissolved in PEG-400 to obtain a concentration of 4 mg/ml. A vehicle without perindopril was also prepared. Perindopril or vehicle drugs were injected subcutaneously into the fracture site once on the day of fracture and once a day for 7 days thereafter. Each injection contained 0.4 mg/kg of perindopril in the experimental group and an equal volume (about 30–50 µl) of vehicle solution in the vehicle groups. In this study, perindopril was injected via the method described previously [20].

### Radiography analysis

At the end of the 2- and 4-week post-fracture observation periods, the rats were anesthetized (10% chloral hydrate, same dosage as above), and ventro-dorsal radiographs of the healing tibiae were taken. X-rays were taken by a DMR+ Mo target mammography machine (22 kV, 250 mAs, GE, USA). The callus diameter was measured in relation to the tibia diameter (%).

### Biomechanical analysis

For biomechanical analysis, the right tibiae were resected and carefully freed from soft tissue, and the intramedullary pins were removed. The bones (n = 6 per group at each time point) were then subjected to a three-point bending test using a commercial material testing system (Instron 4302; Instron, Norwood, MA, USA) as previously reported [20], [21]. Briefly, the tibia was placed in the material testing machine on two supports separated by a distance of 1.5 mm, and the testing area was defined as the central part of the callus. A compression load was applied at a rate of 2.0 mm/min until breakage. From the load–deflection curve recorded by a connected computer, the ultimate load at failure (N; maximum force that the tibia could bear), total energy absorption (MJ; energy absorbed by the tibia during compression), and stiffness (N/mm; slope of the load-deflection curve from the linear part) were calculated.

### Histological analysis

Immediately after animals were killed, the right tibia was harvested without damaging the periosteum. Extreme care was taken to not traumatize the callus. After the steel wire had been withdrawn, the middle part of the tibia that contained the callus was cut. Specimens (n = 6 per group at each time point) were then fixed in 4% buffered formaldehyde for approximately 48 h at room temperature and decalcified in ethylene diaminetetra-acetic acid (EDTA, 0.5 mol/L, PH 7.4) for approximately 4 weeks. The EDTA was refreshed every 3 days until a fine needle could easily be inserted into the bone. Decalcified specimens were then washed, dehydrated in gradient alcohol, embedded in paraffin wax, and cut into 4-µm-thick sections along the longitudinal plane of the tibia. The sections were stained with hematoxylin and eosin and another section was stained with a modified Ponceau trichrome stain, and the slices were examined qualitatively under light microscopy (Leica Instruments GmbH, Germany) with digital cameras.

### Micro-CT analysis

For the micro-CT scan, a desktop micro-CT system (GE Healthcare, USA) was employed. All right tibiae (n = 6 per group at each time point) were prepared by cutting them into 10-mm-long blocks with the callus included and then storing them in 70% ethanol. These specimens were secured with a foam board to prevent them from shifting during scanning at a voltage of 55 kV and a current of 145 µA. Then, 16.4-µm-thick coronal images were reconstructed to evaluate longitudinal fracture callus features. The scanned zone included the original cortical diaphyseal bone and the entire diameter of the periosteal and endosteal callus. Two-dimensional CT images were reconstructed in 1024×1024-pixel matrices using a standard convolution back-projection procedure. Images were stored in 3-D arrays with an isotropic voxel size of 16.4 µm. A constrained 3-D Gaussian filter was used to partly suppress the noise in the volumes. The high and low radio-opacity mineralized tissues were differentially segmented by a two-level global thresholding procedure [21], [22]. The callus volume of interest (VOI) was defined as new-formed bone tissues; the medullary canal volume and the original bone tissue were excluded from evaluation in accordance with previous reports [23], [24]. After segmentation, the following parameters were quantified within the VOI: bone volume over total volume (BV/TV) and average cross-sectional area (CsAr).

### Statistical analysis

All values are expressed as the mean with their standard deviation (mean±S.D.) in the text and the tables. Statistical analyses were conducted using the statistics package SPSS 13.0 (SPSS, Chicago, IL, USA). Differences among treatment groups were tested by one-way analysis of variance (ANOVA). If significant differences were indicated, differences between the means of two groups were tested by Fisher's protected least significant difference (PLSD). Differences were considered significant for p<0.05 and highly significant for p<0.01.
